# THE UNEXPECTED FAMILY CAREGIVER: UNVEILING THE CHARACTERISTICS OF YOUNGER ADULT CAREGIVERS

**DOI:** 10.1093/geroni/igae098.2088

**Published:** 2024-12-31

**Authors:** Katie Spears, Janelle Gore, Erin Bouldin, Akilah Ali, Jenny Walker, Benjamin Olivari, Lisa McGuire

**Affiliations:** Oak Ridge Institute of Science and Education, Georgia, United States; University of Alabama, Alabama, United States; University of Utah, Salt Lake City, Utah, United States; Centers for Disease Control and Prevention, Atlanta, Georgia, United States; Centers for Disease Control and Prevention, Atlanta, Georgia, United States; Centers for Disease Control and Prevention, Atlanta, Georgia, United States; GSA, Atlanta, Georgia, United States

## Abstract

With the growing proportion of older adults in the US population, both the number of persons who will need care and the need for family caregivers will increase. Previous studies have found most caregivers to be middle-aged or older adults, but due to a widening gap in care needs and caregivers available, the importance of younger adult caregivers is growing. This analysis uses Behavioral Risk Factor Surveillance System data from the optional Caregiver Module administered in 2021-2022 in 47 states and Puerto Rico. We described the health characteristics of young adults aged 18-34 (n=45,627), who we classified as current caregivers, anticipated caregivers (expected to provide care in the next 2 years), and non-caregivers. We calculated weighted estimates and made comparisons using chi-square tests. Caregivers reported significantly higher prevalence of obesity (34.3%), depression (35.2%), and current cigarette smoking (17.2%) compared to anticipated caregivers and non-caregivers. Caregivers also reported a higher prevalence of frequent mental distress (14 days or more of poor mental health in the past month) (32.5%) compared to anticipated caregivers and non-caregivers. Most caregivers provided less than 9 hours of care a week (57.8%, 95% CI=54.7-60.9) and helped with personal care tasks (51%, 95% CI=47.9-54.1). Caregivers had more risk factors for chronic disease and poorer mental health than those not currently or anticipated to provide care. Public health efforts can recognize and include younger caregivers in their efforts to maximize physical and mental health for all caregivers to assure care recipients are well-supported.

